# Spreading New Light on Attention Restoration Theory: An Environmental Posner Paradigm

**DOI:** 10.3390/brainsci15060578

**Published:** 2025-05-27

**Authors:** Alessandro Piedimonte, Gianluca Lanzo, Francesco Campaci, Valeria Volpino, Elisa Carlino

**Affiliations:** 1 Department of Neurosciences “Rita Levi Montalcini”, University of Turin, Corso Raffaello 30, 10126 Turin, Italy; alessandro.piedimonte@unito.it (A.P.); gianluca.lanzo@unito.it (G.L.); francesco.campaci@unito.it (F.C.); valeria.volpino@unito.it (V.V.); 2 Carlo Molo Foundation, Via della Rocca 24bis, 10123 Turin, Italy

**Keywords:** Attention Restoration Theory, environmental neuroscience, environmental psychology, attention, Posner paradigm

## Abstract

**Background/Objectives**: Environmental psychology has long investigated how exposure to natural versus urban environments influences cognitive processes, particularly attention. According to Attention Restoration Theory (ART), natural scenes promote involuntary attention and facilitate recovery from mental fatigue. In this study, we used a modified Posner cueing paradigm to assess how natural and urban backgrounds affect both exogenous (involuntary) and endogenous (voluntary) attention. To capture both behavioral and neural responses, the study collected reaction times (RTs) as a measure of task performance, alongside electrophysiological data (event-related potentials, ERPs: P1, N1, P2, N2, and P3) to explore underlying attentional processes. **Methods**: Participants completed a visuospatial task in which visual cues anticipated the appearance of a target stimulus, while background images depicting either natural or urban environments remained visible throughout. Attention was assessed under both valid (cue correctly predicts target location) and invalid (cue misleads target location) conditions. **Results**: The overall findings align with the existing literature: RTs were shorter in valid trials compared to invalid ones. No main facilitation effect from natural backgrounds was observed. However, participants showed slower RTs in invalid trials with natural backgrounds, which may support ART by suggesting that attention restoration could lead to slower responses in certain attentional scenarios. Electrophysiological data reinforced these behavioral results, revealing an increased N2 amplitude in the natural background invalid condition. **Conclusions**: Despite some limitations, this study provides novel insights into human–nature interactions, offering a fresh perspective on the complex relationship between environment and cognition.

## 1. Introduction

Human cognition, behavior, and emotional states are continuously influenced by a complex interplay of internal and external factors [1]. In recent years, the relationship between individuals and their surrounding environment, particularly the natural world, has gained renewed scientific interest, largely in response to increasingly urbanized lifestyles. As most of the global population now resides in urban areas [2], a trend that is expected to grow in the coming decades [3], the cognitive demands of daily life have intensified [4], prompting investigation into how different environmental contexts affect mental functioning, work performance, and lifestyle.

The psychological impact of physical environments—both natural and urban—has been extensively explored since the 1960s, culminating in what Pol (2007) [5] describes as the “green shift” of the late 20th century: a cultural and scientific turn toward sustainability and ecological awareness. Within this context, two prominent theoretical frameworks have emerged. Stress Reduction Theory (SRT) emphasizes the emotional and physiological calming effects of natural settings [6] while Attention Restoration Theory (ART) focuses on the cognitive benefits, particularly the restoration of directed attention following mental fatigue [7,8]. ART posits that natural environments foster involuntary attention, also referred to as soft fascination, which requires minimal cognitive effort and allows fatigued directed attention systems to recover [4,8,9,10]. In cognitive neuroscience, *attention* refers to the set of processes by which certain information is selectively prioritized for processing, while other inputs are suppressed. This includes both voluntary (endogenous) and involuntary (exogenous) mechanisms that modulate sensory and cognitive activity depending on task demands and environmental salience [11,12].

Empirical research has largely supported ART. Studies have shown that exposure to natural environments improves cognitive performance, particularly on tasks requiring sustained attention [13,14,15,16,17,18,19]. For instance, students with access to natural views from their dormitories exhibited enhanced attentional capacities [19], while elderly individuals and adolescents demonstrated greater cognitive restoration in natural compared to urban settings [20,21,22,23]. However, despite these findings, the specific interplay between natural environments and different attentional subsystems remains partially unexplored. Notably, some studies have questioned whether increased environmental fascination necessarily translates to improved directed attention [24].

To address this gap, the present study examines how environmental context influences both exogenous (involuntary) and endogenous (voluntary) attention. Specifically, we employed a modified Posner cueing paradigm [12] in which participants were presented with either natural or urban background scenes during a visuospatial attention task. This paradigm is well-suited to dissociate attentional components: exogenous attention is rapidly and automatically triggered by peripheral cues, while endogenous attention is consciously directed and involves top-down processing [11]. Participants’ behavioral responses were assessed through reaction times (RTs), measured as the latency to identify a target stimulus following valid or invalid cues. In addition to behavioral data, we recorded event-related potentials (ERPs) to investigate the neural correlates of attentional processing within different environmental contexts. This approach builds on previous work showing that natural and urban scenes elicit distinct patterns of visual evoked potentials [25,26,27,28]. ERP components such as the N1 and N2 are sensitive to attentional shifts and may reflect differential engagement of cognitive resources in response to environmental cues [29,30,31]. The N1 is an early negative deflection occurring around 115–175 ms post-stimulus, associated with selective sensory processing, while the N2 emerges around 200–280 ms and is often linked to conflict monitoring and cognitive control mechanisms [32].

Based on ART and prior empirical findings, we hypothesized that natural environments would facilitate attentional performance, leading to faster RTs compared to urban settings. Furthermore, we expected ERP markers to reveal distinct neural signatures associated with attentional modulation by environmental context. By integrating behavioral and electrophysiological data, this study aims to contribute to a deeper understanding of how exposure to natural versus urban environments shapes attentional dynamics, offering new insights into the neurocognitive mechanisms underlying human–nature interactions. To write the paper, Microsoft Word 365 (https://www.microsoft.com/it-it/microsoft-365/free-office-online-for-the-web, accessed on 25 May 2025), version 2406 (build 17726.20160) was used.

## 2. Materials and Methods

### 2.1. Sample Size and Power Analysis

A priori power analysis was conducted using G*Power 3.1 software to determine the appropriate sample size for this study, which employed a mixed-factor 2 × 2 × 2 repeated measures ANOVA design. The within-subject factors were Environment (Urban vs. Green), Posner Condition (Endogenous vs. Exogenous), and Trial Validity (Valid vs. Invalid). Based on previous research indicating a medium effect size in similar contexts, we set the effect size to Cohen’s f = 0.35 [33,34]. The analysis was conducted with an alpha level of 0.05 and a power level of 0.95 to ensure a high probability of detecting significant effects while minimizing the risk of Type II errors.

The power analysis indicated that a total sample size of 13 participants would be sufficient to achieve the desired power (actual power = 0.96). Therefore, we determined that our study design is adequately powered to detect meaningful differences, aligning with the standards in the field of environmental psychology.

### 2.2. Participants

A total of 15 healthy volunteers (7 females and 8 males, mean age = 25.9 ± 2.2) participated in this study. Participants were selected from the student population of the University of Turin (age < 30), with no history of medical conditions (e.g., epilepsy or visual deficits). All participants signed a written informed consent form before attending the test. Participants were informed that they would take part in a study investigating visual perception and attention using the Posner paradigm [12,35]. The study protocol was approved by the Ethics Committee of the University of Turin as following the Declaration of Helsinki principles.

### 2.3. Experimental Procedure

Participants were seated comfortably in an armchair positioned 20 inches (50 cm) from a monitor displaying the visual stimuli, a viewing distance commonly employed in ERP studies to ensure optimal central fixation and minimize eye movement artifacts [36,37]. They were instructed to place their right hand on the keyboard, with the index finger resting on the left arrow key and the ring finger on the right arrow key. Hand positioning was standardized to maintain a fixed association between stimulus location and motor response, minimizing variability in response execution across trials. The task required participants to detect and respond as quickly as possible to a target visual stimulus, which was presented under two distinct conditions: endogenous and exogenous. The target stimulus consisted of a stylized dartboard, featuring three concentric circles colored red–white–red. The cue stimulus varied depending on the attentional condition, following the classic Posner paradigm.

#### 2.3.1. Endogenous Condition

The endogenous condition requires top-down attentional modulation to interpret the directional cue [12,35,38,39]. Top-down attention is voluntary and goal-directed, relying on internal cues and expectations to guide behavior [11,12]. In this condition, the cue was a centrally presented white arrow pointing toward the likely location of the upcoming target with 80% validity. This cue required participants to interpret its direction, engaging top-down attentional processes. The sequence of visual events was as follows: (i) a fixation cross displayed for 400 ms; (ii) the cue stimulus presented for 100 ms; and (iii) a fixation cross reappearing for 100 ms, followed by the target stimulus.

#### 2.3.2. Exogenous Condition

The exogenous condition, characterized by brief cue presentations and target-shaped cues, engages automatic, bottom-up attention mechanisms, as demonstrated in EEG studies. Bottom-up attention is automatic and stimulus-driven, triggered by salient external changes in the environment [11,12]. In this condition, the cue was a circle identical in size to the target. In valid trials, this cue appeared in the same location as the subsequent target; in invalid trials, it appeared in the opposite, mirrored location. The sequence began with (i) a fixation cross; (ii) after 400 ms, the cue stimulus appeared for 50 ms, indicating the likely target location with 80% accuracy; and (iii) 50 ms after cue offset, the target stimulus was presented.

In both conditions, participants responded to the appearance of the target by pressing the left or right arrow key, depending on its location. The final fixation and target stimuli remained on screen until the participant responded. Reaction times (RTs) were defined as the interval between the onset of the target stimulus and the participant’s key press.

To enrich the visual context, background images depicting natural or urban environments—obtained from Google Street View (www.google.com/streetview, accessed on 20 March 2024)—were used in both conditions. To maintain experimental control and to reduce stimulus variability with its possible impact on low-level features processing, we used four representative images: two depicting natural environments and two depicting urban environments. This methodological choice is supported by prior research demonstrating that even brief exposure to single natural scenes can elicit measurable attentional benefits, in line with predictions from ART (Kaplan & Kaplan, 1989; Berto, 2005; Berman et al., 2008 [7,9,33]). Using a limited set of well-controlled stimuli allowed us to isolate the specific effects of environmental context on attentional processes while minimizing potential confounds introduced by heterogeneous image properties. Each attentional condition consisted of 60 trials, balanced across natural and urban backgrounds, resulting in 60 trials with a natural scene and 60 with an urban scene per condition. The trial order was counterbalanced across participants.

**Figure 1:**
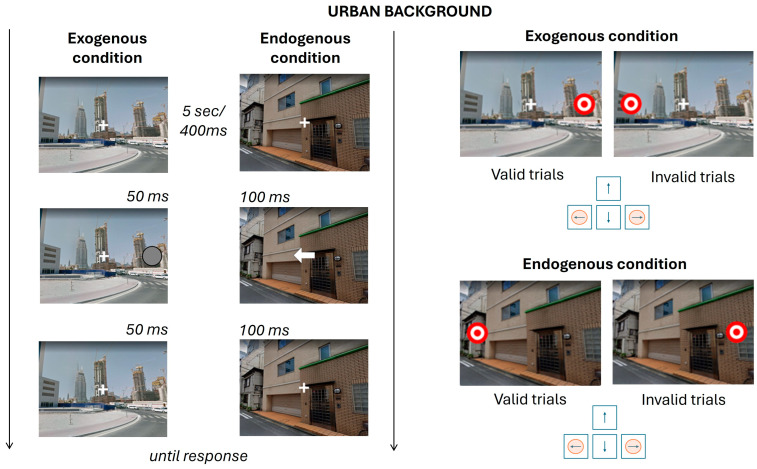
Presentation of all conditions for the urban background. The figure summarizes the timing of the experimental trials. On the left, the sequence of events for cue presentation is shown; on the right, the possible outcomes for target location and corresponding keypress responses are illustrated.

**Figure 2:**
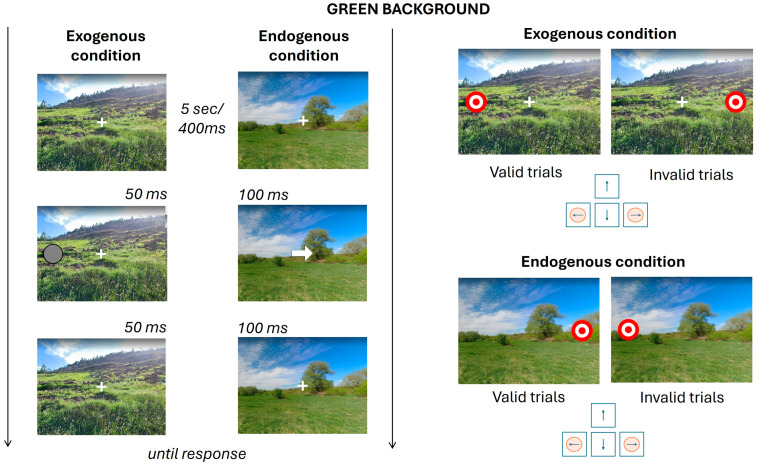
Presentation of all conditions for the green background. The figure summarizes the timing of the experimental trials. On the left, the sequence of events for cue presentation is shown; on the right, the possible outcomes for target location and corresponding keypress responses are illustrated.

Figure 1 and Figure 2 provide an overview of all possible task conditions. In Figure 1, conditions with the green background are subdivided according to the Posner attentional paradigm, exogenous and endogenous, and further subdivided into valid and invalid trials. In Figure 2, the same structure is applied to conditions featuring an urban background, following the same two-step classification: attentional condition (exogenous vs. endogenous) and trial validity (valid vs. invalid). The timing associated with each segment of the task has been reported. At the beginning of each condition, the fixation cross is presented for 5 s. Every trial consists of a fixation cross displayed for 400 ms, the cue stimulus, lasting 50 ms in exogenous conditions and 100 ms in endogenous ones, and another fixation cross presented for the same duration as the cue. Finally, the target stimulus appears and remains on screen until the participant provides a response. The Posner paradigm was designed using PsychoPy [40].

### 2.4. Electroencephalography

During the experiment, electroencephalographic (EEG) data were recorded using a 61-electrode cap arranged according to an augmented 10–20 system (Galileo, EBNeuro S.p.a., Florence, Italy), with the reference electrode positioned at CPPz and the ground electrode at FCz. Throughout data acquisition, electrode impedance was maintained below 5 kΩ for all active channels. EEG signals were digitized at a sampling rate of 1024 Hz. Data preprocessing was carried out in MATLAB R2023a (MathWorks Inc., Natick, MA, USA) using the EEGLAB toolbox [41]. Continuous EEG recordings were first band-pass filtered between 1 and 30 Hz using a finite impulse response (FIR) filter, in order to enhance the performance of subsequent independent component analysis (ICA, [42]). The EEG data were then segmented into 700 ms epochs, spanning from −200 ms to +500 ms relative to the onset of the target stimulus in each trial. Each dataset yielded a total of 240 epochs: 120 for the endogenous condition and 120 for the exogenous condition. Each of these sets was further divided into 60 epochs per background type (natural or urban). Within each group of 60 trials, 48 were valid trials and 12 were invalid trials. As a result, eight distinct average waveforms were computed for each participant, corresponding to each trial type: valid endogenous trials with natural and urban backgrounds; valid exogenous trials with natural and urban backgrounds; and the same combinations for invalid trials. Eye movement and muscle artifacts were removed through independent component analysis (ICA, [42]). Specifically, independent components (ICs) associated with ocular and muscular activity were automatically identified and eliminated using the ICLabel plugin for EEGLAB [43]. Components classified as eye or muscle activity with a confidence level equal to or greater than 90% were removed. Baseline correction was then applied by subtracting the mean voltage during the −200 to 0 ms pre-stimulus interval. Epochs exhibiting amplitudes exceeding ±100 μV were excluded from further analysis. Finally, the remaining artifact-free epochs were averaged for each condition and participant. Given the variability in the cortical areas investigated across different visual paradigms (for a recent review, see [44]), visual evoked potential (VEP) averages were computed over a posterior region of interest that included both parieto-occipital (PO) and occipital electrodes, as previously suggested [45,46]. Specifically, the electrodes PO3, PO4, PO7, PO8, POz, O1, O2, and Oz were included in the analysis. VEP components were identified based on their characteristic latencies following target onset. The following temporal windows were used: P1 (60–140 ms), N1 (115–175 ms), P2 (145–240 ms), N2 (200–280 ms), and P300 (250–310 ms) [47,48]. These components were selected based on their established relevance in the literature on visual attention and cognitive control. The early components (P1 and N1) are sensitive to perceptual encoding and attentional orienting [29,47,49,50,51,52,53], while later components (P2, N2, and P3) are commonly associated with conflict detection, executive control, and decision-related processing [47,48,54,55,56,57]. This selection allowed us to capture the full temporal dynamics of attentional modulation during the cueing task.

### 2.5. Behavioral Data Analysis

The behavioral data analysis was conducted to examine differences in reaction times (RTs) across the various conditions of the experimental paradigm. In particular, the analysis focused on RT variations between valid and invalid trials, across endogenous and exogenous Posner conditions, and between natural (green) and urban background environments, as well as their interactions. Differences in mean RTs were assessed using a 2 × 2 × 2 mixed-factor repeated-measures ANOVA, with all factors defined within subjects. The three within-subject factors were: Background Environment (Urban vs. Green), Posner Condition (Endogenous vs. Exogenous), and Trial Validity (Valid vs. Invalid). For clarity, these factors will hereafter be referred to as Environment, PCond, and Validity, respectively. When significant interactions were observed, post hoc comparisons were performed using post hoc Bonferroni correction to adjust for multiple testing.

### 2.6. Electrophysiological Analysis

Similarly, to behavioral data, differences in mean VEPs were tested by 2 × 2 × 2 mixed factors ANOVAs with Environment, PCond, and Validity all as within factors. To explore significant interactions, a post hoc Bonferroni correction was applied for multiple testing.

For all analysis, the level of significance was set at *p* < 0.05, and data in the figures are presented as the mean ± standard error of the mean (SEM).

## 3. Results

### 3.1. Behavioral Results

Behavioral results, i.e., RTs of each condition, are summarized in Table 1.

**Figure 3:**
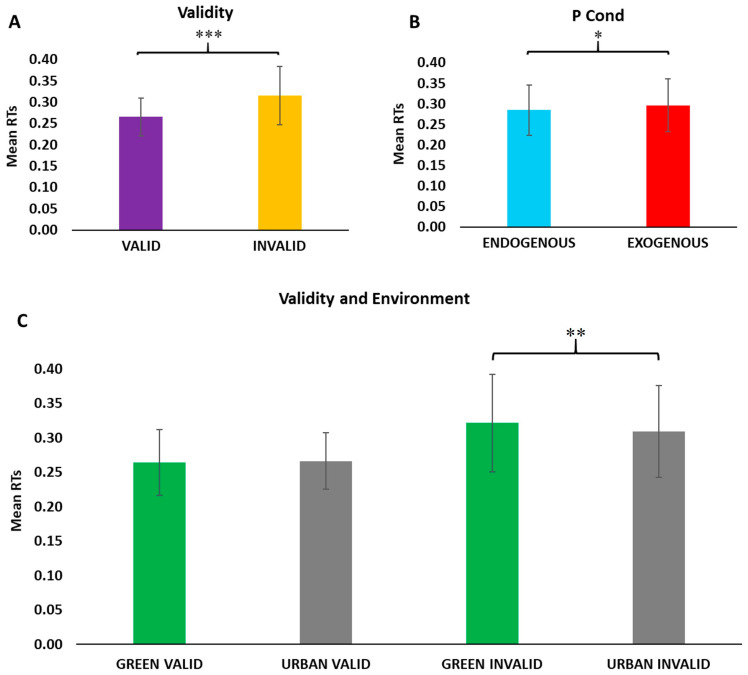
Main Behavioral results. In (**A**), the differences between overall Validity trials; in purple, the Valid trials, and in yellow, the Invalid trials. In (**B**), the differences between overall Posner trials; in light blue, the Endogenous trials, and in red, the Exogenous trials. In (**C**), the differences between trial Validity and background Environment; in green, the Green trials, and in gray, the Urban trials. *Y*-axis indicates the Reaction Times. Error bars represent SEM; * *p* < 0.05, ** *p* < 0.01, *** *p* < 0.001.

ANOVA analysis shows a main effect of Validity [F(1, 14) = 54.440, *p* = 0.00000], depicting a significant facilitation effect for Valid trials compared to Invalid trials (Figure 3A). Moreover, the main effect of PCond was observed [F(1, 14) = 4.7506, *p* = 0.04686], showing faster RTs for the Endogenous trials compared to Exogenous ones (Figure 3B). No main effect for Environment has been observed. Moreover, the analysis showed a significant interaction between Validity and Environment [F(1, 14) = 9.1060, *p* = 0.00922]. After Bonferroni post hoc correction, the analysis showed faster RTs for Invalid trials with an Urban background compared to Invalid trials with a Green background (Figure 3C).

### 3.2. Electrophysiological Results

#### 3.2.1. VEP Analysis for Validity, PCond, and Environment

**Figure 4:**
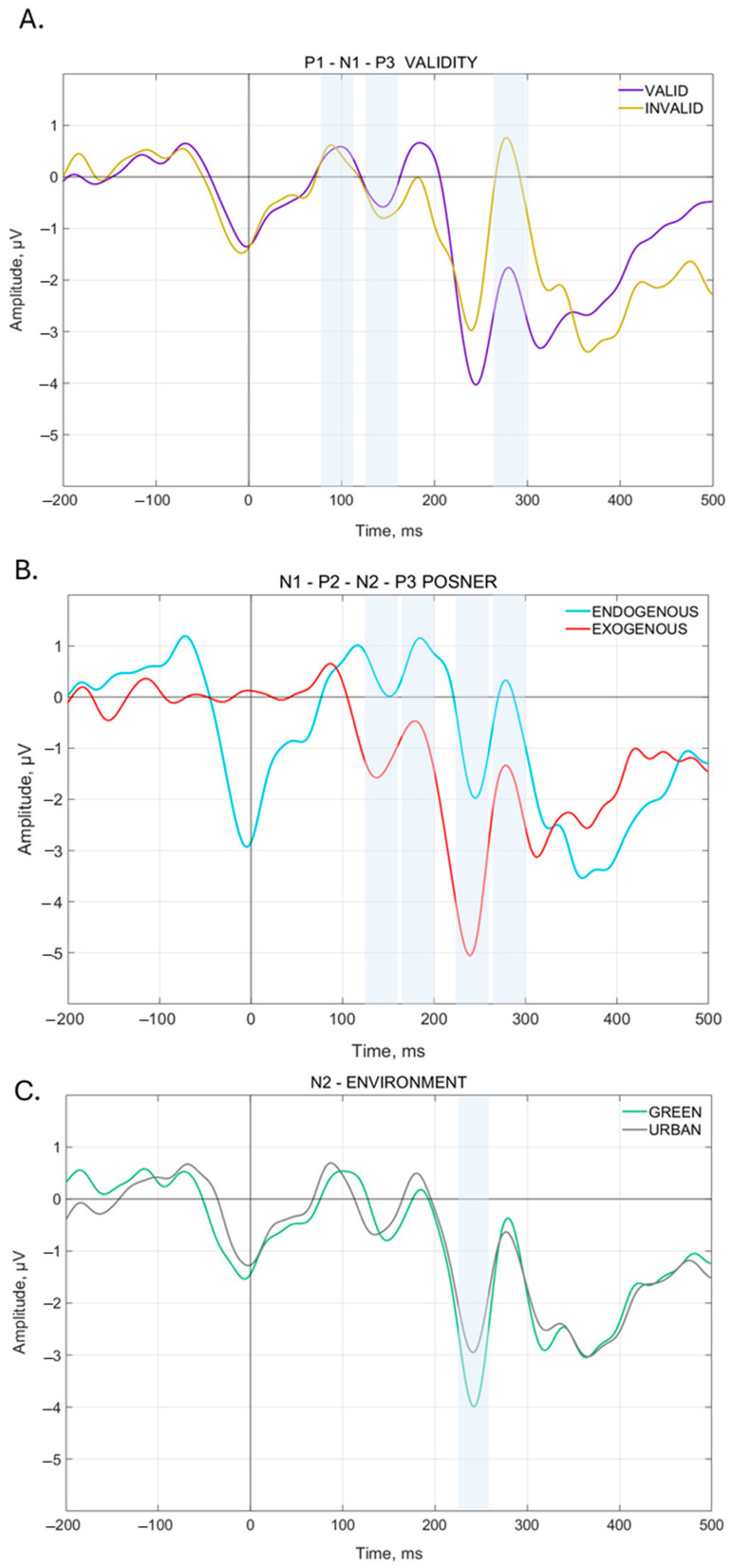
Main Electrophysiological results. In (**A**), the overall VEP results for Validity (valid–invalid). In purple, the valid trials, in yellow, the invalid trials. Invalid trials show a higher amplitude compared to valid ones in all three components, P1, N1, and P3. In (**B**), the overall VEP results for PCond (endogenous–exogenous). In red, the exogenous condition, in light blue, the endogenous one. Exogenous trials showed a higher amplitude compared to endogenous ones in negative components (N1 and N2). Endogenous trials showed a higher amplitude compared to exogenous ones in positive components (P1 and P3). In (**C**), the overall VEP results for Environment (natural–urban). In green, the natural trials, in gray, the urban ones. *Y*-axis indicates the VEPs Amplitude (µV). *X*-axis indicates Time (ms).

The analysis on the VEP mean amplitude showed a significant main effect of Validity for the N1 [F(1, 14) = 9.6919, *p* = 0.00763], P1 [F(1, 14) = 9.1242, *p* = 0.00917], and P3 [F(1, 14) = 33.946, *p* = 0.00004] components. Specifically, for each ERP, the analysis showed a significantly higher amplitude during Invalid trials compared to Valid ones. A main effect of PCond has been observed for N1 [F(1, 14) = 36.915, *p* = 0.00003], N2 [F(1, 14) = 21.304, *p* = 0.00040], P2 [F(1, 14) = 24.424, *p* = 0.00022], and P3 [F(1, 14) = 12.794, *p* = 0.00303]. In particular, the analysis showed a significant increase in amplitude for N1 and N2 during Exogenous trials compared to the Endogenous ones, and for P2 and P3 during Endogenous trials compared to the Exogenous ones. Finally, a main effect of Environment has been observed in N2 [F(1, 14) = 5.5219, *p* = 0.03397] with a significantly higher amplitude for Green background environments compared to Urban ones. These main effect results are summarized in Figure 4.

#### 3.2.2. N1 Component

**Figure 5:**
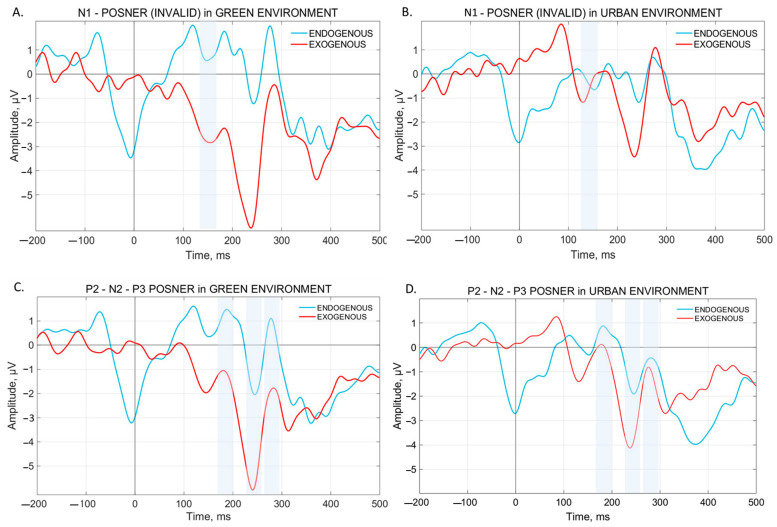
Specific VEPs Electrophysiological results for Posner Condition. In red, the Exogenous trials, in light blue, the Endogenous trials. In (**A**), the significantly higher N1 amplitude for Green Exogenous trials compared to Green Endogenous ones. In (**B**), the lack of differences for N1 between Urban Exogenous and Endogenous trials. In (**C**), from left to right, the significantly higher P2 amplitude for Green Endogenous trials compared to Green Endogenous trials, the significantly higher N2 amplitude during Green Exogenous than Green Endogenous trials, and the significantly higher P3 amplitude for Green Endogenous trials compared to Green Endogenous trials. In (**D**), from left to right, the lack of differences in P2 amplitude for Urban Endogenous trials compared to Urban Endogenous trials, the significantly higher N2 amplitude during Urban Exogenous than Urban Endogenous trials, and no differences in P3 amplitude for Urban Endogenous trials compared to Urban Endogenous trials. *Y*-axis indicates the VEPs Amplitude (µV). *X*-axis indicates Time (ms).

The analysis of N1 showed a significant interaction Environment × PCond × Validity [F(1, 14) = 6.2882, *p* = 0.02509]. The Bonferroni post hoc test showed significant changes in N1 amplitude only with a Green background. Specifically, N1 amplitude is significantly higher during Green Invalid Exogenous trials compared to Green Invalid Endogenous trials. The N1 pattern of this analysis is shown in Figure 5A. Additionally, for the N1 component, there is also a slight tendency in the Environment × PCond interaction [F(1, 14) = 4.0092, *p* = 0.06501] and, in the Bonferroni post hoc test, significant differences emerged between Green Endogenous trials and Green Exogenous trials, but not between Endogenous and Exogenous trials with Urban background.

#### 3.2.3. P1 Component

The analysis of P1 showed a significant interaction between Environment × PCond [F(1, 14) = 6.2588, *p* = 0.02538] and a slight tendency for Environment × PCond × Validity [F(1, 14) = 4.1571, *p* = 0.06081], but neither survived Bonferroni correction.

#### 3.2.4. N2 Component

**Figure 6:**
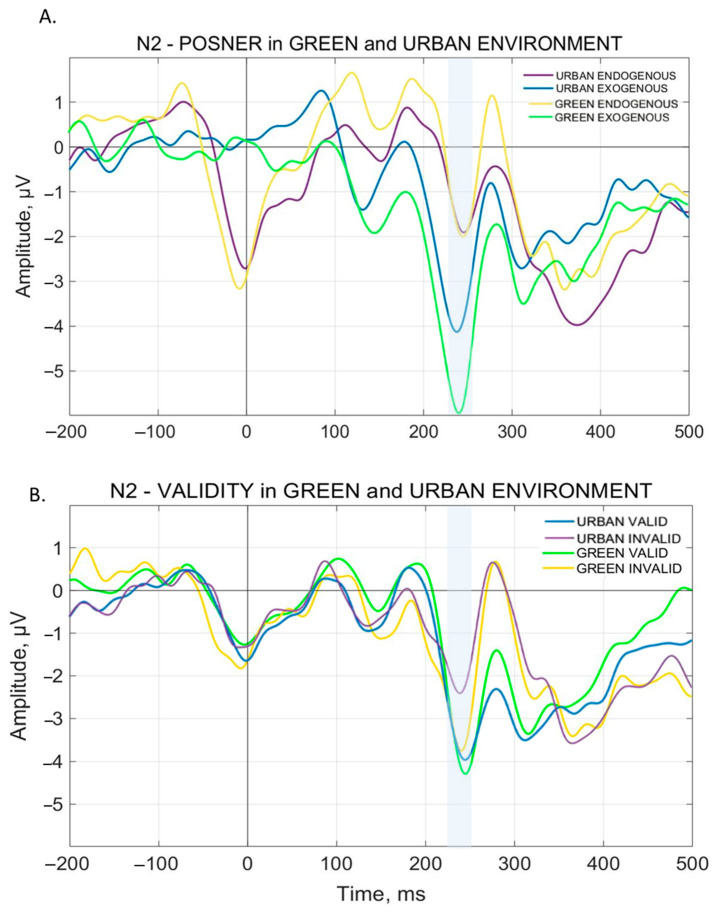
Specific VEPs Electrophysiological results for Posner Condition and Validity in Green and Urban environments. Darker colors, blue and purple, stand for Urban environments, while lighter colors, green and yellow, stand for Green environment. In (**A**), the significantly higher N2 amplitude during Green Exogenous trials than all other conditions. In (**B**), the significantly lower N2 amplitude for Urban Invalid trials compared to Green Invalid trials and Urban Valid trials. *Y*-axis indicates the VEPs Amplitude (µV). *X*-axis indicates Time (ms).

The analysis of N2 reveals a significant interaction both for Environment × PCond [F(1, 14) = 9.4362, *p* = 0.00828] and Environment × Validity [F(1, 14) = 5.4597, *p* = 0.03484]. The Bonferroni post hoc test showed a higher N2 amplitude for Urban Exogenous trials compared to Urban Endogenous trials. This is shown in Figure 5D. Furthermore, N2 amplitude during Green Exogenous trials is significantly higher than all the other related conditions, i.e., Green Endogenous trials and Urban Exogenous trials. This is shown in Figure 5C and Figure 6A. For Environment × Validity interaction, N2 amplitude results significantly lower for Urban Invalid trials compared to Green Invalid trials and Urban Valid trials. Surprisingly, no significant interaction between Green Valid trials and Green Invalid trials has been observed. This is shown in Figure 6B.

#### 3.2.5. P2 Component

The analysis of P2 reveals a significant interaction Environment × PCond [F(1, 14) = 6.7475, *p* = 0.02108]. The Bonferroni post hoc test shows that P2 amplitude for Green Endogenous trials is higher compared to Green Endogenous trials. No differences were found between Urban Endogenous and Exogenous trials. This is shown in Figure 5C,D.

#### 3.2.6. P3 Component

The analysis of P3 shows a significant interaction Environment × PCond [F(1, 14) = 10.003, *p* = 0.00691]. Bonferroni correction does not show significant differences between the PCond for Green or Urban Environments and reveals only significant differences between Green Exogenous trials and Green Endogenous trials, with higher P3 amplitude for the latter, while no differences are present between Urban Exogenous trials and Urban Endogenous trials. This is shown is Figure 5C,D.

The authors supplied all behavioral and electrophysiological measures (means and standard deviations) online contained in Excel S1.

## 4. Discussion

The present study aimed to investigate how environmental context, specifically natural versus urban visual backgrounds, modulates attentional performance and its neural correlates. To this end, we employed a modified version of the Posner cueing paradigm, allowing us to disentangle endogenous (voluntary) and exogenous (involuntary) attention under different environmental conditions. Behavioral performance was assessed through reaction times (RTs), while neural activity was measured using EEG event-related potentials (ERPs).

Overall, the modified Posner paradigm elicited the expected behavioral effects: reaction times were significantly slower in invalid trials compared to valid ones, confirming the classic validity effect typical of Posner tasks. ERP components analysis mimic behavioral findings, revealing higher P1, N1, and P3 amplitudes in invalid trials compared to valid ones, consistent with an increase in attentional and cognitive demands. While P1 and N1 patterns differ from classical findings—where these components are typically enhanced for valid trials [29,58], P3 component shows higher amplitude during invalid trials, consistent with novel stimulus processing and contextual updating [47,48,55,56,57]. In our data, P1 and N1 differences may reflect motor ERP influences.

Interestingly, having established that our paradigm elicits cognitive modulation during stimulus processing, we then found that environmental context adds an additional layer of modulation, affecting both behavioral and physiological outcomes. In fact, RTs were significantly slower for invalid trials presented with the natural background compared to the urban one, and the N2 component for these trials perfectly mirrored behavioral results, showing a higher amplitude for natural backgrounds, thus suggesting an enhanced cognitive control and conflict monitoring in this condition [59,60,61].

Our findings both align with and diverge from prior research on the cognitive effects of natural environments, thereby offering new nuance to the existing literature. As of now, a substantial body of research supports the assumption that natural environments enhance attentional performance, highlighting the cognitive benefits of exposure to natural landscapes, particularly for directed attention [9], working memory [62], and recovery from mental fatigue [33]. These studies support the idea that natural environments enhance attentional functioning through mechanisms such as perceptual fluency and attentional restoration. Our hypotheses followed these roots and were grounded in ART concepts [7].

According to ART, natural environments engage involuntary attention (“soft fascination”), allowing for the restoration of directed attentional resources and facilitating cognitive performance. However, in our study, we observed slower RTs and increased N2 amplitudes specifically during invalid trials with natural backgrounds, when attentional disengagement and reorienting were required. These findings suggest that the supposed automatic involuntary attentional engagement, driven by natural context, may interfere with rather than support top-down goal-directed attentional control, possibly due to increased distraction or perceptual salience of natural scenes. In other words, we propose that the attentional system may become “captured” by the natural scenes, reducing the availability of resources for efficiently re-orienting attention.

Interestingly, this framework highlights that cognitive resources are limited, resonating with the cognitive control model of work-related flow [63], which proposes that effort and volitional control are required for experiencing and efficient flow. From this perspective, during the interference created by the Posner task, attentional resources could already be engaged by the aesthetic and perceptual salience of natural scenes, leaving fewer resources for reorienting attention and performing the task accurately.

Alternatively, our results may align with motor inhibition theories, particularly considering the “stopping for knowledge” hypothesis [64]. This view proposes that aesthetically engaging stimuli, such as natural landscapes, can inhibit motor responses by diverting cognitive resources toward sensory processing, thereby shifting the brain’s priority from action to exploration [32,64,65]. Within our paradigm, when participants had to suppress interference during Posner invalid trials, the greater sensory salience of natural backgrounds could have additionally increased cognitive load, specifically for perceptual processing. Hence, the added conflict of attentional resources inhibited motor readiness, leading to the observed behavioral and physiological modulations. Our findings suggest that attentional performance in natural environments encompasses a complex interplay between perceptual engagement, cognitive control, and motor preparation, extending the perspective beyond ART alone.

In a recent study, Grassini et al. (2022) found that exposure to natural environments promoted the presence of alpha waves in the EEG correlates, which are notoriously considered a marker of relaxation [66]. This result could well integrate the restoration of attentional resources from ART and support our findings of enhanced neural activity during the exploration of natural scenes. In a previous study, Grassini et al. (2019) proposed lower attentional demands for natural scenes compared to urban ones [26]. It is possible that during a fast attentional task, cognitive resources become attracted and stored for exploration of the natural scene, the brain shifts to alpha waves, and it is more challenging to re-engage with task demands, even more so when cognitive conflict is elicited by Posner invalid trials.

Interestingly, as our findings seem to be in the opposite direction of the ones from Grassini’s research group, it is important to stress that we employ a different paradigm, specifically to test these effects during an attentional task, and we discuss primarily the effects on the N2 component during cognitive conflict. In fact, we found that, in particular conditions, N2 in the urban environment could be modulated by our paradigm, consistent with Grassini’s.

Nevertheless, our study differs from previous investigation of ART concepts with behavioral and electrophysiological measures: here, attentional performance is not evaluated before and after the exposure to natural environments, in a classical pre/post fashion, and instead, it is embedded in a dynamic context, where natural and urban environments are presented in real time during the attentional task and evaluation. This approach may account for the discrepancies with the literature, to the extent to which no other study presented salient natural stimuli during the unfolding of a highly demanding attentional task. On top of this, our study employs electrophysiological measures, allowing for high-resolution analysis of the temporal dynamics of attention modulation. This dual-level (behavioral and neurophysiological) investigation offers a richer picture of how natural and urban environments affect both performance and underlying brain mechanisms.

Nevertheless, several limitations need acknowledgment: sample size is relatively modest, although statistically powered, potentially limiting generalizability, stimuli presentation included only two images per environment type, raising questions on whether results are driven by the actual environment type or stimuli-specific features, such as luminance, color distribution, and spatial structure. Future work should explore a broader and more diverse range of natural settings, such as lakes, forests, or mountains, to disentangle these possibilities.

To expand our findings, different techniques should be considered to investigate these phenomena, such as combining ERP measures with eye-tracking, pupillometry, or motor kinematics may further elucidate the sensory–motor trade-offs underlying attentional shifts in natural versus urban contexts. For instance, recent work reported a reduction in oculomotor activity when participants viewed natural scenes versus urban ones [67], underlying again the complex interplay between attentional resources and natural context. Additionally, planning this research employing immersive tools, such as augmented reality (AR) and virtual reality (VR), should also be considered to confirm and enhance the experiment findings.

From a translational point of view, the so-called “green environments” are a proven source of well-being and improvements in different health outcomes [68], both physical and psychological. Green environments, lessening physiological stressors [69], and in line with our proposal, inhibiting motor responses, while enhancing perception and exploration, could be integrated into cognitive rehabilitation therapies. Different patients with different conditions (such as ADHD, frontal lesions, OCD, and Parkinson’s syndrome) could benefit from the facilitation of cognitive load through the presentation of naturalistic stimuli paired with specific task stimuli. For instance, patients with frontal lesions struggle to inhibit motor responses to the go/no–go task, interfering with required attentional shifts [70,71]. In those situations, unnecessary motor responses could be delayed or suppressed, potentially permitting the storage of attentional resources directly on task demands, performing better, and in a broader picture improving rehabilitation course.

Lastly, these findings could expand to the construction of urban spaces, and could be viewed as a confirmation that, effectively, the implementation of plants, gardens, benches, water pools, bright colorful items, and the overall prevalence of vegetation over hardscapes elements [72] does not just modify overt perception and judgments, but can also alter physiological markers of attention and perception, albeit implementing this paradigm in salient and ecological context is needed to confirm that real environments can elicit specific cerebral changes.

## Figures and Tables

**Table 1:** Behavioral results corresponding to RTs of each subject in each condition.

**Subject**	**Green**					
	**Exogenous**			**Endogenous**		
	***Mean RT***	***Mean Valid RT***	***Mean Invalid RT***	***Mean RT***	***Mean Valid RT***	***Mean Invalid RT***
*1*	0.249137024	0.240151741	0.285078154	0.257391674	0.241511387	0.320912823
*2*	0.334997077	0.324027356	0.378875963	0.306402855	0.29073568	0.369071553
*3*	0.314887324	0.302259423	0.365398927	0.270287685	0.25885686	0.316010983
*4*	0.246914843	0.242665717	0.263911347	0.284231804	0.275132427	0.320629315
*5*	0.245307665	0.237926057	0.274834098	0.262065705	0.247940076	0.318568218
*6*	0.208870532	0.20893939	0.208595102	0.162348879	0.157734365	0.180806933
*7*	0.308951705	0.283142809	0.412187291	0.326095302	0.319530306	0.352355286
*8*	0.300592355	0.280657166	0.380333109	0.284099017	0.269768323	0.34142179
*9*	0.328078283	0.307884736	0.408852468	0.330228997	0.31762009	0.380664627
*10*	0.286731258	0.277493841	0.323680927	0.270894826	0.259204828	0.317654821
*11*	0.277554035	0.263471209	0.333885339	0.283397209	0.270130053	0.336465833
*12*	0.304645934	0.280679632	0.400511143	0.295713726	0.288500067	0.324568361
*13*	0.229191206	0.220708353	0.263122622	0.222430795	0.214108593	0.255719607
*14*	0.396158187	0.38003358	0.460656614	0.325574109	0.311220268	0.382989475
*15*	0.192131213	0.191224892	0.195756499	0.173118983	0.172210673	0.176752226
***Means***	*0.281609909*	*0.269417727*	*0.33037864*	*0.270285438*	*0.2596136*	*0.31297279*
**Subject**	**Urban**					
	**Exogenous**			**Endogenous**		
	***Mean RT***	***Mean Valid RT***	***Mean Invalid RT***	***Mean RT***	***Mean Valid RT***	***Mean Invalid RT***
*1*	0.245175936	0.241321198	0.260594888	0.254439064	0.24780198	0.280987399
*2*	0.332952607	0.323645963	0.370179181	0.278007826	0.262958739	0.338204173
*3*	0.287662048	0.278070822	0.326026954	0.292104551	0.280773914	0.337427101
*4*	0.258678978	0.253121243	0.280909916	0.268210498	0.265306041	0.279828326
*5*	0.268228474	0.263271148	0.288057774	0.276304924	0.274288303	0.284371408
*6*	0.183940172	0.181863903	0.192245249	0.202987578	0.197332076	0.225609583
*7*	0.30550634	0.294627381	0.349022175	0.314165882	0.305839286	0.347472265
*8*	0.287193544	0.264362553	0.378517512	0.315462459	0.299863006	0.37786027
*9*	0.328048234	0.313167996	0.387569183	0.317294426	0.306452926	0.360660425
*10*	0.286416865	0.286912626	0.284433822	0.257804996	0.250971788	0.285137825
*11*	0.284355723	0.272209128	0.332942103	0.25881389	0.247810467	0.302827583
*12*	0.313099547	0.300254705	0.364478916	0.275954405	0.266226896	0.314864444
*13*	0.246286015	0.238789332	0.276272744	0.238854801	0.235539774	0.252114909
*14*	0.337127176	0.320183819	0.404900604	0.357536685	0.335300835	0.446480088
*15*	0.217737504	0.216865175	0.221226818	0.157133369	0.162206261	0.136841799
***Means***	*0.278827277*	*0.269911133*	*0.314491856*	*0.271005024*	*0.262578153*	*0.304712507*

## Data Availability

The original contributions presented in the study are included in the article, further inquiries can be directed to the corresponding author.

## Supplementary Materials

The following supporting information can be downloaded at: https://www.mdpi.com/article/10.3390/brainsci15060578/s1.
